# Comparing the accuracy between shear wave elastography and strain elastography in the diagnosis of breast tumors: Systematic review and meta-analysis

**DOI:** 10.1097/MD.0000000000029139

**Published:** 2022-05-13

**Authors:** Huayu Wu, Shengnan Zhang, Cong Wang, Yumei Yan

**Affiliations:** a The First Affiliated Hospital of Dalian Medical University, Dalian, China; b Ultrasound department of the First Affiliated Hospital of Dalian Medical University, Dalian, China.

**Keywords:** breast tumors, shear wave elastography, strain elastography, meta-analysis

## Abstract

**Background:**

Shear wave elastography and strain elastography are two new ultrasonic techniques developed rapidly in recent years. Changes in tissue elasticity occur after normal tissue changes. Elastography technique transforms the elastic information of tissue into optical information for display. Thus more intuitive display of tissue elasticity. Due to the differences in principles and related imaging parameters between the two elastic imaging methods, and the acquisition and interpretation of image data in strain elastic imaging method largely depends on the experience of inspectors, and due to the significant differences between the techniques of inspectors, As a result, conflicting results have been obtained in different scholars’ studies on the accuracy comparison of the two elastography techniques in the diagnosis of breast tumors. This meta-analysis aims to compare the accuracy of the two elastography methods in the diagnosis of breast tumors, so as to provide more accurate diagnostic means for patients with breast tumors. The final results will show which elastography method is more accurate in the diagnosis of breast tumors, reduce unnecessary biopsies and provide a reference for clinical decision making.

**Methods:**

We will examine published and unpublished randomized controlled trials, observational studies and abstracts without publication type or language restrictions, and search relevant literatures in PubMed, Web of Science, Wanfang Database, CNQI and other databases until December 30, 2020. The authors will independently search relevant literature records, scan titles and abstracts, full text, collect data and assess the risk of bias. Data will be analyzed by using Meta Disc1.4 software and Stata14.0 software. Heterogeneity tests and combined sensitivity, specificity, positive and negative likelihood ratio, diagnostic odds ratio, and area under the summary receiver operating characteristic curve will be performed by using Meta Disc1.4 software. Stata14.0 software will be used for sensitivity analysis and publication bias test.

**Results:**

The results of this systematic review will demonstrate the accuracy of the two elastography methods in the diagnosis of breast tumors.

**Discussion:**

The results will provide useful evidence for the comparison of the diagnostic accuracy of shear wave elastography and strain elastography in breast tumors.

**Other:**

This study was not funded. Register name: PROSPERO. Registration number: CRD42021251110.

## 1 Introduction

Breast cancer is the most common malignant tumor in women and the main cause of most cancer-related deaths. The main reason for the high mortality rate of breast cancer is distant organ metastasis.^[1]^ Early breast cancer without obvious clinical symptoms, often manifested as painless sclerosis, easy to be ignored.^[2]^ Therefore, early screening is particularly important. Traditional screening methods for breast cancer include mammography and ultrasound (US), which are commonly used in clinical screening for breast cancer. US are a common imaging method for the diagnosis and prognosis of breast tumors, but traditional US still has limitations such as false positive, and elastography has been proved to improve the specificity of breast mass evaluation.^[3]^ Elastography is a new ultrasonic technique developed rapidly in recent years, which can accurately determine the hardness of tissues. Benign and malignant breast lesions have different hardness on palpation for a long time, and lesions with low activity are more likely to be malignant.^[4]^ Therefore, this technology can be widely used in breast cancer screening. Elastography including shear wave elastography (SWE) and strain elastography (SE) in two ways, however, they are quite different in imaging methods. SWE can evaluate soft tissue elasticity quantitatively, and SWE is through the detection of acoustic radiation pulse, continuous is focused on the vibration of the lesions are observed in the organization, thus appearing transverse shear wave. The shear wave travels along a plane perpendicular to the radiation pulse applied by the sensor, picking up the acoustic radiation from the transducer. Basing on the shear wave velocity of accurate quantitative testing organization, elastic numerical measurement organization, it can effectively distinguish benign and malignant mammary gland with good reproducibility and objectivity. However, artifacts caused by reflection and refraction may increase due to the large variation in shear wave velocities in normal and abnormal breast tissues. SE is to apply pressure to the tissue according to the different elastic coefficients between different groups, so as to make the deformation degree of the tissue under different stress levels under the action of stress and deformation of the operator tissue. The amplitude of the pressure echo signal changes into a real-time color image before and after the movement, and the color of the image reflects the hardness of the tissue. Finally, the determined tissue elasticity is color-coded and superimposed on a b-mode image on an ultrasound device monitor.^[5–8]^ However, this technique requires manual compression of the sensor, which may result in higher measurement variations in the compression process, it is difficult to measure the magnitude of force or stress, and it is impossible to calculate the absolute elasticity. By using strain ratios, we can reduce the influence of different external compression forces, thus improving the comparability of data.^[9]^ When SWE and SE are applied to the same breast lesion, they will yield consistent or similar results if examined by an experienced operator. However, because each elastography technique has its inherent defects, lead to false positive or negative result, according to the application of the technique and its diagnostic criteria, there may be different.^[10]^ The result of a study has shown that the thickness of the breast can affect the relationship between the two kinds of elastography diagnosis, when the lesion location deep, will reduce the strain type elastic imaging diagnostic accuracy, SWE is not affected. This study also mentioned that SWE and SE have different sensitivity and specificity for different breast thickness, and even different histopathology and tumor grade.^[11]^ However, Mirinae Seo^[12]^ scholar found no difference in the sensitivity and specificity of SE and SWE for lesions, and the diagnostic results of the two elastography methods were indeed contradictory. We hope that the results of this study will provide value for the accurate diagnosis of breast tumors in order to improve the sensitivity and specificity of the evaluation of breast masses. Breast lesions detected by routine US can be classified according to the possibility of malignancy in the Breast Imaging-Reporting and Data System, but routine US can lead to false positive rates and thus unnecessary biopsies. The existence of elastic imaging combined with the use of US can reduce Breast Imaging Reporting and Data System 4A lesions, reduce unnecessary biopsy of benign breast lesions,^[13,14]^ reduce patients’ anxiety and medical costs, without reducing the sensitivity of breast cancer diagnosis.

## 2 Materials and methods

The study was followed by PROSPERO. Registration number: CRD42021251110, the preferred reporting item of the Systematic Review and Meta-Analysis was the guideline for the design of this study.

### 2.1 Type of study

Only high-quality clinical cohort or case-control studies will be included in this study. The Chinese and English literatures on the comparison of SWE and/or SE in the diagnosis of breast tumors will not be regionally limited and case reports, animal studies, expert experience, conference articles and duplicate publications will be excluded.

### 2.2 Type of patients

The patients should be those who have experienced breast tumors, which have been confirmed by postoperative pathological biopsy, regardless of pathological type, unilateral or bilateral, regardless of age.

### 2.3 Intervention and comparison

This study compared the diagnostic value of SWE and SE on the basis of pathological diagnosis.

### 2.4 Type of outcomes

Primary outcomes include combined sensitivity, specificity, positive likelihood ratio and negative likelihood ratio, diagnostic odds ratio (DOR), and area under curve (AUC).

### 2.5 Search methods

From the establishment of PubMed, Web of Science, Wanfang Database and China national knowledge infrastructure Database until December 30, 2020, relevant literatures will be searched and research data will be obtained without language restrictions. We will use professional retrieval in Wanfang Database, and the search strategy is as follows: “The theme: (Breast Tumor or Breast Cancer) and the theme: (Shear wave elastography and SE) and the theme: (Diagnosis)”. PubMed's search strategy is shown in the Table 1. Other databases will be used for the same strategy.

**Table 1:** PubMed search strategy sample.

Search terms:
1. Breast cancer or breast neoplasm or neoplasm, breast or breast tumors.
2. Shear wave elastography and strain elastography
3. Sensitive or sensitivity and specificity or predictive or predictive value of test or accuracy
4. and 1-3

### 2.6 Data extraction and quality assessment

The authors will independently select trials based on inclusion criteria and import the literature into NoteExpress software. Duplicate or unqualified studies are then deleted. Quality assessment is very important in the systematic review of diagnostic accuracy research. Quality Assessment of Diagnostic Accuracy Studies tool developed by Whiting ^[15]^ will be used to evaluate the Quality of the included Studies. The tool is an evidence-based quality assessment tool for a systematic review of diagnostic accuracy studies, it can assess the quality of studies on diagnostic accuracy, and it is able to distinguish between high quality and low quality studies. The tool includes 14 items and provides guidelines for scoring each item included in the tool, and the quality of studies will be assessed by these items from the tool. Each question should be answered “yes” (2), “no” (1), or “unclear” (0). Answer “yes” if there was sufficient information from the study fits one of the items, “no” if not. If there was insufficient information available to make a judgement then it should be answered as “unclear”. Finally, we decided to choose 14 Diagnostic test Quality evaluation criteria. Quality Assessment of Diagnostic Accuracy Study scores range from 0 to 28, with a score of 22 indicating good quality. Any differences between the two investigators will be resolved by a third investigator through discussion or negotiation, and the authors will independently select trials and remove duplicate or incompatible studies based on inclusion criteria. The following data will be extracted from each included study: first author's last name, age, sample size, number of lesions, subject origin, instrumentation, gold standard, and diagnostic accuracy. True positive, true negative, false positive, and false negative data will be collected simultaneously.

### 2.7 Statistical analysis

The combined sensitivity, specificity, positive likelihood ratio, negative likelihood ratio, DOR, and area under curve will be calculated by meta-Disc 1.4 software. The DOR of a test is the ratio of the positivity of the disease to the positivity of the non-disease, or the DOR can be read as the ratio of the chance of testing positive to the chance of testing negative. The value of DOR ranges from 0 to infinity. The larger the value is, the better the performance of identification test is. The threshold effect was determined by observing whether the image was distributed in shoulder or arm shape or whether the *P* value was greater than .05. If *P* > .05 exists threshold effect, summary receiver operating characteristic curve can only be fitted and the area under summary receiver operating characteristic curve can be calculated. If *P* < .05, *I*^2^ < 50%, there was no heterogeneity caused by non-threshold effect, and the fixed effect model was used to merge the effect size. If *P* < .05, *I*^2^≥50%, indicating that there was heterogeneity caused by non-threshold effect. Random effect model was used to combine effect size. Stata14.0 software will be used for sensitivity analysis and publication bias test. If there was heterogeneity caused by non-threshold effect, further meta-regression analysis should be conducted on the included studies to explore the source of heterogeneity between studies. The Beggs funnel plot and Eggers linear regression test will be used to investigate publication bias.

### 2.8 Ethics and dissemination

We will not obtain ethic documents because this study will be conducted based on the data of published literature. We expect to publish this study in a peer-reviewed journal.

## 3 Discussion

Despite significant advances in cancer research in recent years, breast cancer remains a major health problem and the most common cancer affecting women worldwide, with a significant increase in morbidity and mortality expected in the coming years.^[16]^ Therefore, early detection and early treatment are particularly important. The combination of elastography and ultrasound grayscale has been proved to improve the diagnostic accuracy of breast lesions.^[3,17]^ But which kind of elastography has higher diagnostic accuracy is higher, because of the related literature reports, the argument leads to the clinician in selecting imaging examination method is lack of accurate basis, this study adopts the method of Meta analysis, larger sample sizes, reduce the random error, it will provide the high quality of evidence-based medicine, In the evaluation indexes of the diagnostic experiment obtained through statistics, the combined AUC represents the effectiveness of the examination. AUC > 0.9 indicates high efficiency, medium efficiency 0.7–0.9, and low efficiency 0.5–0.6.^[18]^ By comparing the combined AUC values of the two kinds of elastic imaging, it can be concluded that, In the same way, we will compare the combined sensitivity and specificity of the two imaging methods to determine which elastography has higher accuracy in the diagnosis of breast tumors. SWE can display quantitative information of lesions and surrounding tissues in real time in the form of color superposition. Because shear wave velocity can be measured and is related to Young's modulus (kPa), SE is more dependent on the professional knowledge of the operator, so the expected statistical result should be: The accuracy of shear wave elastography in breast tumor diagnosis is better than SE. We hope that the results of this study will provide a reference for clinical decision making.

## Author contributions

**Conceptualization:** Huayu Wu, Yumei Yan.

**Data curation:** Huayu Wu.

**Formal analysis:** Yumei Yan, Shengnan Zhang.

**Investigation:** Huayu Wu, Shengnan Zhang.

**Methodology:** Cong Wang.

**Resources:** Shengnan Zhang.

**Software:** Huayu Wu.

**Supervision:** Yumei Yan.

**Writing – original draft:** Huayu Wu.

**Writing – review & editing:** Huayu Wu, Yumei Yan.
